# Room-temperature super-extraction system (RTSES) optimizes the anxiolytic- and antidepressant-like behavioural effects of traditional *Xiao-Yao-San* in mice

**DOI:** 10.1186/1749-8546-7-24

**Published:** 2012-11-07

**Authors:** Shih-Hsi Yin, Ching-Cheng Wang, Tain-Junn Cheng, Chia-Yu Chang, Kao-Chang Lin, Wei-Chih Kan, Hsien-Yi Wang, Wenny Mei-Wen Kao, Yen-Liang Kuo, Jian-Chyi Chen, Shun-Lai Li, Chia-Hui Cheng, Jiunn-Jye Chuu

**Affiliations:** 1 Institute of Manufacturing Information and Systems, National Cheng Kung University, Tainan, Taiwan; 2 Department of Neurology, Chi Mei Medical Center, Tainan, Taiwan; 3 Department of Occupational Medicine, Chi Mei Medical Center, Tainan, Taiwan; 4 Department of Occupational Safety, College of Environment, Chia Nan University of Pharmacy and Science, Tainan, Taiwan; 5 Department of Occupational and Environmental Medicine, National Cheng Kung University, Tainan, Taiwan; 6 Department of Food Science and Biotechnology, National Chung Hsing University, Taichung, Taiwan; 7 Institute of Biotechnology, College of Engineering, Southern Taiwan University of Science and Technology, Tainan, Taiwan; 8 Department of Nephrology, Chi-Mei Medical Center, Tainan, Taiwan; 9 Department of Medical Laboratory Science and Biotechnology, Chung Hwa University of Medical Technology, Tainan, Taiwan; 10 Institute of Plant Biology, National Taiwan University, Taipei, Taiwan; 11 MesoPhase Technologies Inc, Tainan, Taiwan; 12 Department of Biotechnology, Southern Taiwan University of Science and Technology, No. 1, Nantai St., Yung-Kang, Tainan, Taiwan

## Abstract

**Background:**

*Xiao-Yao-San* (XYS) is a Chinese medicinal formula for treating anxiety and depression. This study aims to evaluate the use of a room-temperature super-extraction system (RTSES) to extract the major active components of XYS and enhance their psycho-pharmacological effects.

**Methods:**

The neuroprotective roles of XYS/RTSES against reserpine-derived neurotoxicity were evaluated using a glial cell injury system (*in vitro*) and a depression-like C57BL/6 J mouse model (*in vivo*). The anxiolytic-behavioural effects were measured by the elevated plus-maze (EPM) test and the antidepressant effects were evaluated by the forced swimming test (FST) and tail suspension test (TST). Glucose tolerance and insulin resistance were assayed by ELISA. The expression of 5-HT_1A_ receptors in the prefrontal cortex was examined by western blotting.

**Results:**

XYS/RTSES (300 μg/mL) diminished reserpine-induced glial cell death more effectively than either XYS (300 μg/mL) or fluoxetine (30 μM) at 24 h (*P* = 0.0481 and *P* = 0.054, respectively). Oral administration of XYS/RTSES (500 mg/kg/day) for 4 consecutive weeks significantly elevated the ratios of entries (open arms/closed arms; *P* = 0.0177) and shuttle activity (*P* = 0.00149) on the EPM test, and reduced the immobility time by 90% on the TST (*P* = 0.00000538) and FST (*P* = 0.0000053839). XYS/RTSES also improved the regulation of blood glucose (*P* = 0.0305) and increased the insulin sensitivity (*P* = 0.0093). The Western blot results indicated that the activation of cerebral 5-HT_1A_ receptors may be involved in the mechanisms of XYS/RTSES actions.

**Conclusion:**

The RTSES could provide a novel method for extracting effective anxiolytic- and antidepressant-like substances. XYS/RTSES improved the regulation of blood glucose and increased the insulin sensitivity in reserpine-induced anxiety and depression. Neuroprotection of glial cells and activation of cerebral 5-HT_1A_ receptors were also involved.

## Introduction

According to WHO, depression is one of the serious diseases of the past century, and will cause health burdens in 2030 [1], including the development of tension and mental stress as well as the growing incidence of suicide [2]. Depression is a severe psychotic disorder that includes symptoms of depressed mood, sleep disturbance, and psychomotor and body weight abnormalities, among others [3,4]. Recent studies have shown metabolic syndrome abnormalities (obesity, glucose intolerance, hypertension, and hyperlipidemia), that may also be associated features of depression [5,6].

The Chinese medicine formula *Xiao-Yao-San* (XYS) is a mixture of eight crude drugs (*Bupleurum falcatum*, *Angelica sinensis*, *Paeonia lactiflora*, *Atractylodes lancea*, *Wolfiporia cocos*, *Zingiber officinale*, *Mentha arvensis*, and *Glycyrrhiza uralensis*[6]. *Kami-Shoyo-San* (KSS)*, a herbal formula commonly prescribed for climacteric symptoms, which is* derived from some Chinese herbs and similar in composition to XYS in a Japanese Kampo clinic [7,8]. It has been suggested that KSS is a safe and efficacious therapy for relief of climacteric symptoms in postmenopausal women [9]. KSS is commonly used to treat functional dyspepsia, constipation, menopausal symptoms, premenstrual dysphoric disorder, anti-psychotic-induced tardive dyskinesia, panic disorder, parkinsonism, insomnia, and depressive disorders [10-12].

Treatments with XYS had analgesic and antipyretic effects, increased motor activity with a hedonic effect, caused significant reversal of stress-induced deficits in learning and memory in a spatial memory task, and produced anxiolytic-like effects on the elevated plus-maze (EPM) test [13,14]. Accordingly, treatments with KSS significantly lowered the immobility time on the forced swimming test (FST), improved neurogenesis in the hippocampus, and induced antidepressant climbing behaviour, suggesting that XYS and KSS might be effective antidepressant agents [15,16]. KSS increased the plasma tumour necrosis factor-alpha levels in depressed menopausal patients and decreased the interleukin-6 concentrations in women with psychological symptoms [10-12]. It also decreased the homocysteine levels and did not affect the total cholesterol levels in ovariectomized rats [13]. The social interaction test indicated that KSS caused its anxiolytic effect through neurosteroid synthesis followed by gamma-aminobutyric acid/benzodiazepine receptor stimulations in male mice.

Interestingly, clinical examination of patients with depression revealed a rising prevalence of impaired glucose tolerance including high fasting blood glucose and abnormal oral glucose tolerance test (OGTT) curve, suggesting that the frequency of impaired glucose tolerance increases with abnormal glycometabolism in patients with depression [17].

Chinese herbal medicine compositions are very complex, and the traditional methods for extracting effective components have many disadvantages, such as long cycle processes, insufficient extraction rates and residual organic solvents [18,19]. To increase the extraction rate, several novel extraction methods, including cold-soaked extraction, percolation extraction, reflux extraction, shattering extraction, continuous-reflux extraction, ultrahigh-pressure extraction, ultrasonic extraction, and microwave extraction have been used for effective extraction of the bioactive components and major ingredients from Chinese medicinal plants and Chinese herbal prescriptions [20,21]. The room-temperature super-extraction system (RTSES) is a novel, organic, solvent-free extraction technology for ultrasonic extraction of molecules from solid particles [22]. The system contains an ultrasound-producing element that produces concentrated ultrasonic waves, and acoustic cavities that crush the particles in the fluid. As a result, large molecules in the extract retain their activities, and no organic waste and contaminants are present in the extract or during the extraction course [23]. In particular, the RTSES has a higher extraction yield, lower extraction temperature, and shorter extraction time, involves less power consumption, and preserves the extracts’ bioactivities, making it suitable for the extraction of Chinese medicine components.

It was reported that fluoxetine reduced locomotion in the C57BL/6 J and 129SvEv mouse strains, but not in the BALB/c and DBA/2 mouse strains [24]. In the FST, treatment with fluoxetine (10 and 18 mg/kg/day) increased swimming and reduced immobility [24]. Later research showed that the use of inbred C57BL/6 J mice in a pathological condition and chronic fluoxetine treatment was more accurate for estimating antidepressant efficiency [25].

The emotional impacts of XYS (conventional water extraction) and XYS/RTSES (XYS extracted by the RTSES) were assessed for reserpine-induced anxiety- or depressive-like behaviours compared with fluoxetine. In this study, we used the C6 glial cell line, which was cloned from a rat glioma as a model system, to examine the neuroprotective roles of XYS/RTSES treatments against reserpine-mediated injury (*in vitro*). The anxiolytic and antidepressant effects of these treatments in C57BL/6 J mice were investigated through the EPM, and the FST and tail-suspension test (TST), respectively. The oral glucose tolerance and insulin resistance were assessed to evaluate the influences of the plasma glucose level and variable insulin sensitivity in reserpine-induced performance. The expression of 5-hydroxytryptamine 1A (5-HT_1A_) receptors in the prefrontal cortex of mice was measured by western blotting to compare the efficacies of conventional XYS and XYS/RTSES.

This study aims to investigate the RTSES for extracting anxiolytic- and antidepressant-like substances from XYS and to compare the extraction efficacy of the RTSES with those of other methods.

## Methods

### XYS prescription

The XYS prescription was composed of eight Chinese medicinal materials, including *B. falcatum* (15% w/w), *A. sinensis* (15% w/w), *P. lactiflora* (15% w/w), *A. macrocephala Koidz* (15% w/w), *W. cocos* (15% w/w), *Z. officinale* (15% w/w), *M. arvensis* (6% w/w), and *G. uralensis* (4% w/w). These constituents were provided by Sun Ten Pharmaceutical Co. Ltd. (Taipei, Taiwan). *A. macrocephala Koidz* was used according to Miller-Martini *et al.*[12] and Chen *et al.*[26] instead of *A. lancea* (*Thunb*) [27,28]. The Chinese medicinal materials were pulverized before mixing.

### Chemicals and reagents

Minimum essential medium (MEM), foetal bovine serum, sodium bicarbonate, and 0.05% trypsin-EDTA were purchased from Gibco Ltd. (USA). Streptozotocin and fluoxetine were purchased from Sigma (USA). Mouse monoclonal antibodies against 5-HT_1A_ receptor and β-actin were purchased from Santa Cruz Biotechnology Inc. (USA). Nitrocellulose membranes were purchased from NEN Life Science Products (USA). Mouse insulin and glucose ELISA kits were purchased from Mercodia (Sweden).

### Preparation procedures for two crude extracts

For conventional water extraction, 1 kg of XYS powder was suspended in 10 L of double-distilled water and extracted with hot water by boiling twice for 1.5 h each. The residues were filtered for *in vitro* cell toxicity assays. For the XYS/RTSES procedure, 1 kg of XYS crude powder was suspended in 10 L of pure water and extracted by the RTSES with an extraction temperature of 20–22°C, extraction frequency of 40 kHz, and extraction time of 0.5 h [29]. After the extraction, the size of residual powder particles was determined to be 70–300 nm using a laser particle-size analyser (Zetasizer Nano ZS; Malvern Instruments, UK). The extracted solutions (XYS and XYS/RTSES) were vacuum freeze-dried. Unlike the traditional drying, the vacuum freeze-drying process is free from impurities, and can maintain the original material components and active ingredients without material shrinkage or destruction of cells. The dried powders were dissolved in double-distilled water, and the impurity-free solutions were stored at −80°C until use. The working concentration was determined by the initial weight of the raw materials (g) and the final vehicle volume (mL) after vacuum freeze-drying. The solutions were provided for *in vitro* cell toxicity assays and oral administration at 1 h before the animal tests.

### Cell culture

The rat glioma C6 cell line (ATCC# CCL-107) was purchased from the Food Industry Research and Development Institute (Taiwan). The cells were maintained in MEM, containing nonessential amino acids and sodium pyruvate supplemented with 10% heat-inactivated foetal bovine serum, 2 mmol/L glutamine, 100 U/mL penicillin G sodium, and 100 g/mL streptomycin sulfate (Gibco Laboratories, USA) in 75 T culture flasks. For experiments, the cells were subcultured in microtiter plates in the above culture medium in an incubator (95% air and 5% CO_2_ at 37°C), and the medium was changed every 2 days until analysis.

### Cell proliferation assay

*In vitro* cell proliferation assays were performed to evaluate the effects of XYS (100–300 g/mL), XYS/RTSES (100–300 g/mL), and fluoxetine (30 M) on the glioma C6 cell line [30]. Cells at the exponential growth phase were harvested from the culture flasks by trypsinisation and suspended in fresh medium to a density of 5 × 10^4^ cells/mL. The cell suspensions were dispensed into 96-well microplates at 100 L/well and incubated under 5% CO_2_ at 37°C. After 24 h, the suspensions were removed, and 200 L of XYS (100–300 g/mL), XYS/RTSES (100–300 g/mL), and fluoxetine (30 M) were added, and combined this with pretreatment of reserpine (100 M) at 24 h before exposure. Cell proliferation in the microplates was determined by the MTT assay (optical density measurements at 570 nm) after 72 h of incubation. The cell proliferation activity was expressed as the percentage of MTT counts of treated cells relative to those of control cells treated with double-distilled water.

### Animal experiments

Male C57BL/6 J mice at 4–6 weeks of age (weighing 16–18 g) were purchased from the National Laboratory Animal Center (Taiwan). All animals were maintained in laminar flow cabinets with free access to food and water under specific pathogen-free conditions in facilities approved by the Accreditation of Laboratory Animal Care and in accordance with the Institutional Animal Care and Use Committee (IACUC) of the Animal Research Committee of the Chi-Mei Medical Center (Taiwan). The animals were maintained on a daily 12-h/12-h light/dark cycle, and experiments were carried out between 10:00 h and 17:00 h. Behavioural stress/depression was chemically induced by administration of reserpine (2 mg/kg/day) for 4 consecutive days. XYS (500 mg/kg/day), XYS/RTSES (500 mg/kg/day), or fluoxetine (10 mg/kg/day) was administered orally to the mice for 4 consecutive weeks. The doses of XYS (500 mg/kg/day) and XYS/RTSES (500 mg/kg/day) were two times higher than the maximum recommended human dose of 250 mg/kg/day based on area under the curve (AUC) comparisons. Reserpine was administrated by intraperitoneal injection 30 min before the tests. Drug solutions were prepared just before the injection and given in a volume of 0.1 mL for every 10 g body weight by oral administration and intraperitoneal injection, alternatively. During the experiments, the body weight was recorded weekly and blood samples were drawn weekly from the retro-orbital sinus of the mice to monitor the fasting plasma glucose levels. At the end of the experiments, all mice in the control, fluoxetine, XYS, and XYS/RTSES groups were euthanized for Western blotting.

### EPM Test

The EPM test was performed as described previously [31]. The EPM apparatus was constructed from grey Plexiglas, and consisted of two open arms and two enclosed arms with 15-cm-high transparent walls, which were elevated to a height of 55 cm above the floor. A mouse was placed in the apparatus to start exploring the maze from the central square of the apparatus (facing an enclosed arm) for 10 min. The total time spent in the open and closed arms and the total entries were determined. For data analysis, the ratio of entries into open arms to closed arms was analyzed for the evaluation of anxiolytic activity. Data acquisition and analysis were performed automatically using Image EP software (O’Hara & Co., UK).

### FST

The FST was performed as described previously [32]. Individual mice were forced to swim in an open cylindrical container (25-cm height × 10-cm diameter) filled with water at 24.5–25.5°C up to a height of 10 cm. In our study, the total duration of immobility was evaluated in a 10-min swim session in the FST, which was modified on the basis of a previous study [33]. The mice were considered immobile when they made only the movements necessary to keep their head above water. The test was conducted at 1 h after the last drug treatment, and groups of mice were tested in parallel. Each test was conducted in a quiet and warm environment. A decrease in the duration of immobility was indicative of an antidepressant-like effect.

### TST

The TST was performed as described previously [34]. Mice were observed for 6 min, and the cumulative immobility time (latency to immobility, number of immobile segments, and total time spent immobile) during the final 5-min interval of the test was recorded. The total duration of immobility (in seconds) was measured during the 5-min period. A decrease in the duration of immobility was indicative of an antidepressant-like effect.

### OGTT

After an overnight fast, 3 g/kg of D-glucose was administered orally to reserpine-induced mice and saline-treated control mice. Blood samples were collected from the tail vein of each mouse at 0, 15, 45, 95, and 135 min after the start of the oral glucose administration to measure the fasting blood glucose levels [35].

### Insulin tolerance test (ITT)

After an overnight fast, 3 g/kg of D-glucose was administered orally after intraperitoneal injection of 0.5 U of insulin (Sigma-Aldrich, USA) into reserpine-induced mice and saline-treated control mice. The blood samples were collected from the tail vein of each mouse at 0, 15, 45, 95, and 135 min after the oral glucose administration to measure the fasting blood glucose levels [36]. For the ITT, the percentage decrease in blood glucose from the 0-min time point was calculated.

### Western blotting

At the end of the experiments, the mice in each group were euthanized, and the cerebrum was removed for analysis of the expression levels of depression-like relevant proteins [37]. The cerebrum was prepared in lysis buffer (1% NP-40, 150 mM NaCl, 20 mM Tris–HCl pH 7.5, and protease inhibitors). After incubation on ice for 30 min, the lysates were centrifuged (Centrifuge 5804R; Eppendorf Co. Ltd., USA) at 13,000 × *g* for 30 min. Post-nuclear supernatants were mixed with equal volumes of 2× sample buffer (12.5 mm Tris–HCl pH 6.8, 2% SDS, 20% glycerol, and 0.25% bromophenol blue) and boiled for 5 min. The samples were separated in 10% polyacrylamide gels, and then transferred to nitrocellulose membranes. The membranes were blocked with bovine serum albumin in 0.1 M phosphate buffer saline with Tween, containing 0.2 M Na_2_HPO_4_, 0.2 M NaH_2_PO_4_, 1.5 M NaCl and 0.1% Tween 20 for 2 h at room temperature. After blocking, the membranes were incubated with the anti-5-HT_1A_ antibody diluted 1:500 in wash buffer for 1 h at room temperature, followed by four 10-min washes. The membranes were then incubated with a horseradish peroxidase-conjugated anti-rabbit IgG antibody diluted 1:5,000 in wash buffer for 1 h at room temperature, Horseradish peroxidase was added to visualize antibody-bound target protein on the nitrocellulose membrane. Western blot images were obtained using a LAS-3000 analyzer (FUJIFILM LAS-3000; Fuji Photo Film Co. Ltd., Japan). The signals for β-actin were also evaluated to normalize the protein loading.

### Statistical analysis

All results were presented as the mean ± standard deviation (SD). Differences between groups were evaluated by analysis of variance and post-*hoc* comparisons with the Bonferroni step-down (Holm) correction. All analyses were conducted using Sigma Plot software (version 10.0; SPSS Inc., USA). The dose-dependent relationships were visually determined. *P* values less than 0.05 were considered statistically significant.

## Results

### Cell viability

**Figure 1:**
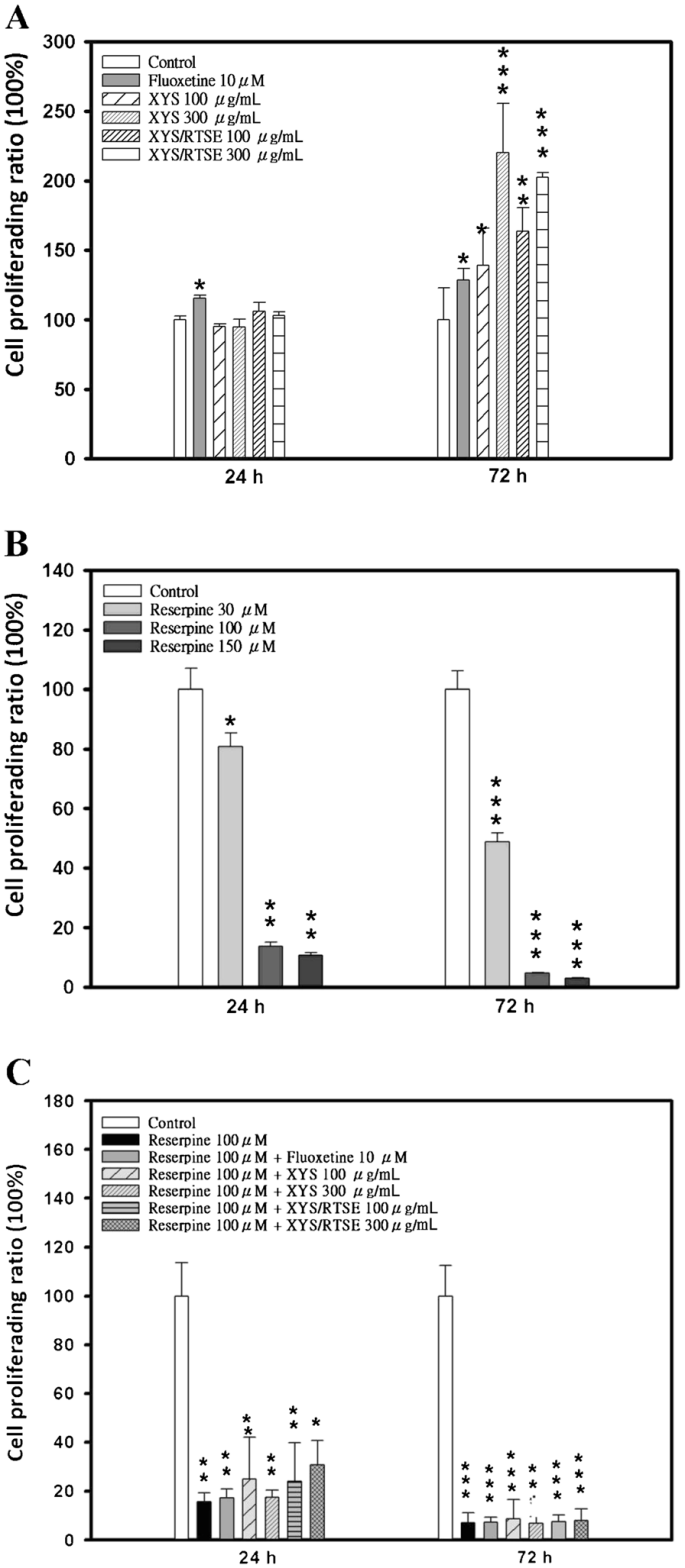
**Cell viability and neuroprotection against reserpine-induced injury.** (**A**–**C**) Using the MTT cell proliferation assay, fluoxetine and different concentrations of XYS and XYS/RTSES were evaluated for cell proliferation (**A**). Various concentrations of reserpine were evaluated for damage to glial cells (**B**). Pretreatment with reserpine (100 M) at 24 h followed by treatment with fluoxetine, XYS, and XYS/RTSES (**C**) on the glioma C6 cell line after incubation for 24 and 72 h is shown. Each value is expressed as the mean ± SD of three culture wells. *P < 0.05, **P < 0.01, ***P < 0.001, significant difference compared with control C6 cells. ^#^*P* < 0.05, ^##^*P* < 0.01, ^###^*P* < 0.001, significantly difference compared with XYS alone.

In the MTT assay, fluoxetine (30 μM) slightly stimulated the cell growth at 24 h (*P* = 0.034) and 72 h (*P* = 0.0479), while XYS and XYS/RTSES showed dose-dependent enhancing effects on the cell proliferation at 72 h (Figure 1A). Reserpine (100 and 150 M) severely affected the cell growth (> 90%) at 24 h (*P* = 0.0012) and 72 h (*P* = 0.0000097) (Figure 1B). When combined with pretreatment of reserpine (100 M), XYS/RTSES (300 μg/mL) prevented reserpine-induced injury at 24 h (*P* = 0.0274), while XYS did not (Figure 1C), demonstrating that XYS/RTSES improved the neurological function.

### Anxiolytic- and antidepressant-like effects

**Figure 2:**
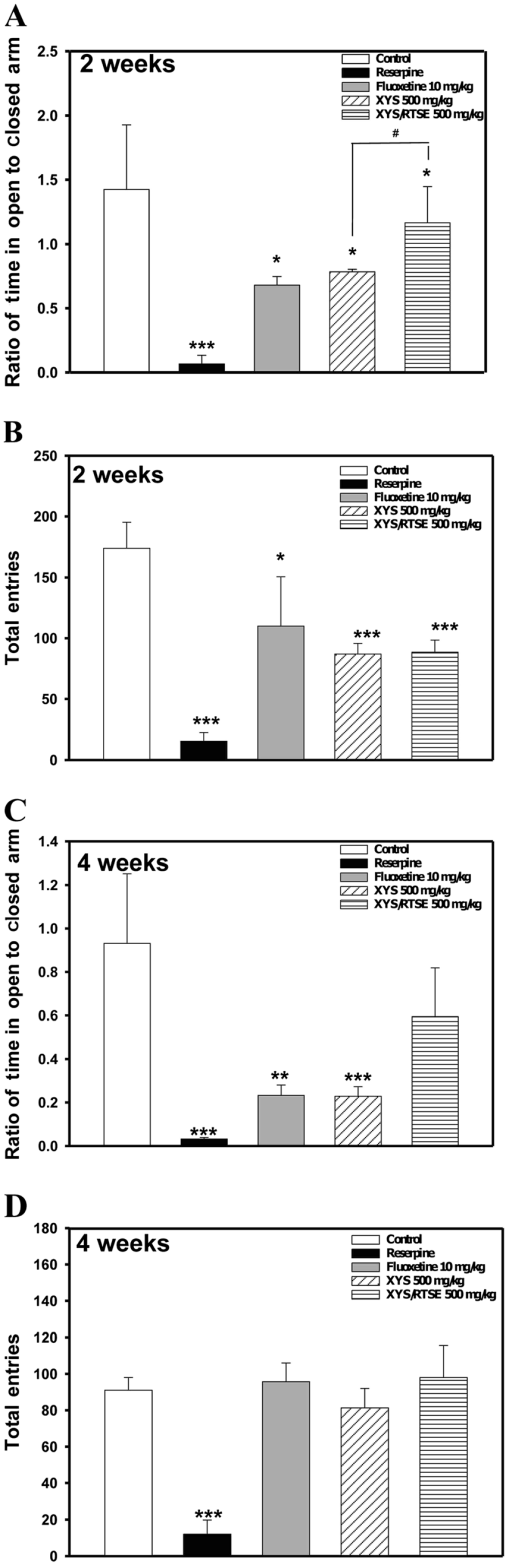
**Anxiety behaviour in the EPM test.** C57BL/6 J mice (N = 8) completed the EPM test. The time spent (in seconds) in the open arms and closed arms during the 10-min test period was evaluated. After treatment with fluoxetine, XYS, and XYS/RTSES, the ratios of entries onto open arms to closed arms were calculated at 2 weeks (**A**) and 4 weeks (**B**), respectively. The numbers of total entries (crossing between open and closed arms) were recorded at 2 weeks (**C**) and 4 weeks (**D**). Each value is expressed as the mean ± SD of 8 mice. *P < 0.05, **P < 0.01, ***P < 0.001, significant difference compared with control C57BL/6 J mice. ^#^*P* < 0.05, ^##^*P* < 0.01, ^###^*P* < 0.001, significant difference compared with XYS alone.

In the EPM test, reserpine (2 mg/kg/day) increased the time spent in the open arms and decreased the time spent in the closed arms after administration for 4 consecutive days, giving smaller ratios of entries onto open arms to closed arms than the control group at 2 weeks (*P* = 0.00008037) (Figure 2A) and 4 weeks (*P* = 0.000029) (Figure 2C). Accordingly, oral administration of XYS/RTSES for 2 consecutive weeks (*P* = 0.037) or 4 consecutive weeks (*P* = 0.05) elevated the ratios of entries onto open arms to closed arms, compared with the same treatment (500 mg/kg/day) of XYS (*P* = 0.018) (Figure 2B). The XYS/RTSES (500 mg/kg/day) group showed reduced locomotor activity (*P* = 0.000102) and no significant difference compared with the normal control mice at 4 weeks (*P* = 0.5565) (Figure 2D). These findings suggest that XYS/RTSES may reduce reserpine-induced anxiety in mice.

**Figure 3:**
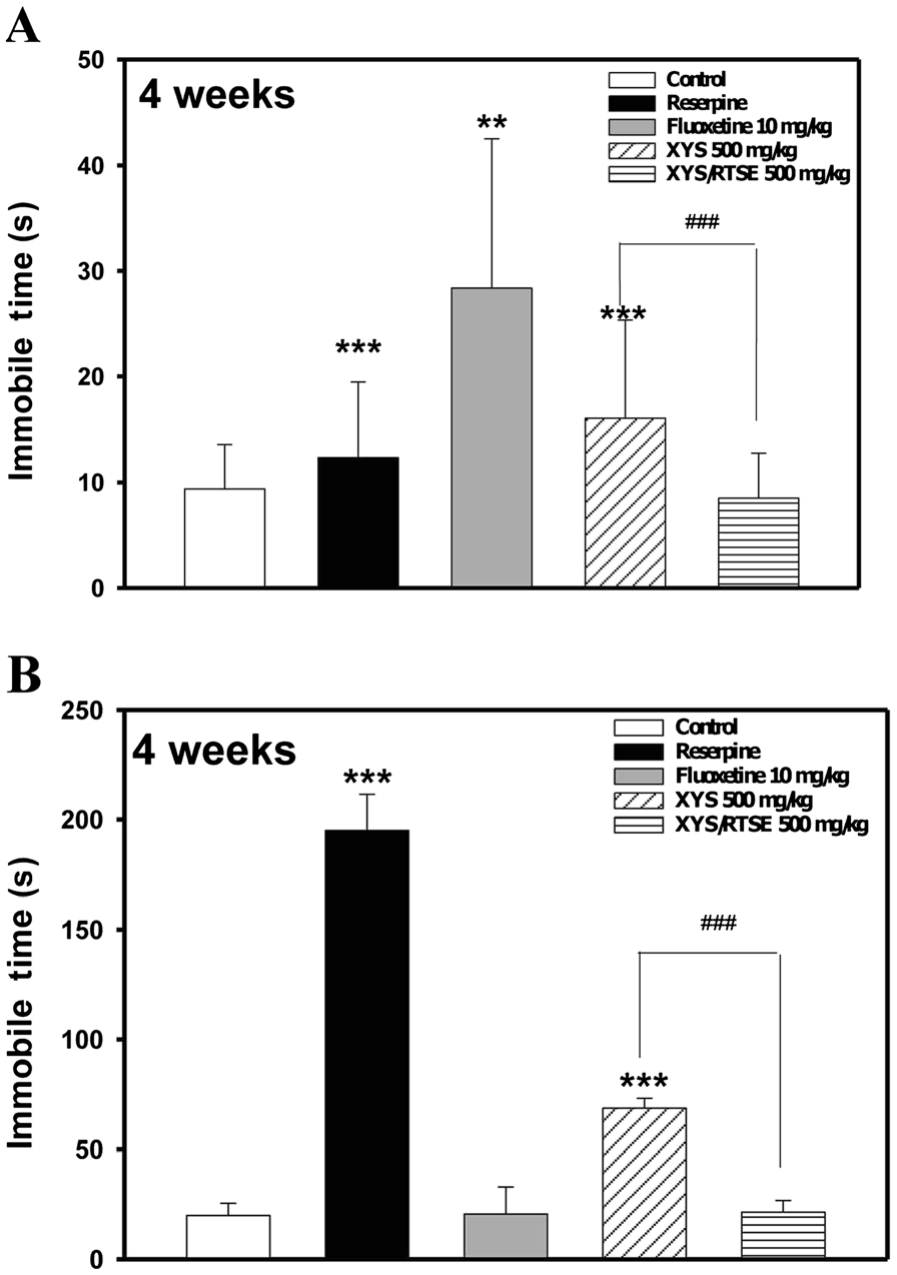
**Depressive-like behaviour.** Studies were conducted in C57BL/6 J mice (N = 8). After 4 weeks of treatment with fluoxetine, XYS, and XYS/RTSES, the total duration of immobility (in seconds) in the mice was measured on the FST (**A**) and TST (**B**). Each value is expressed as the mean ± SD of 8 mice. *P < 0.05, **P < 0.01, ***P < 0.001, significant difference compared with control C57BL/6 J mice. ^#^*P* < 0.05, ^##^*P* < 0.01, ^###^*P* < 0.001, significant difference compared with XYS alone.

The effects of XYS (500 mg/kg/day, peroral) or XYS/RTSES (500 mg/kg/day, peroral), in combination with reserpine (2 mg/kg/day, intraperitoneal) pretreatment, on the total duration of immobility in mice were evaluated at 4 weeks by the FST (Figure 3A) and TST (Figure 3B). In the FST, our data indicated that administration of XYS/RTSES significantly reduced the immobility time by 90% (*P* = 0.000002486), while fluoxetine and XYS reduced the immobility time by 75% (*P* = 0.001157) and 50% (*P* = 0.000437), respectively (Figure 3A). Similar effects were observed in the TST (Figure 3B).

**Figure 4:**
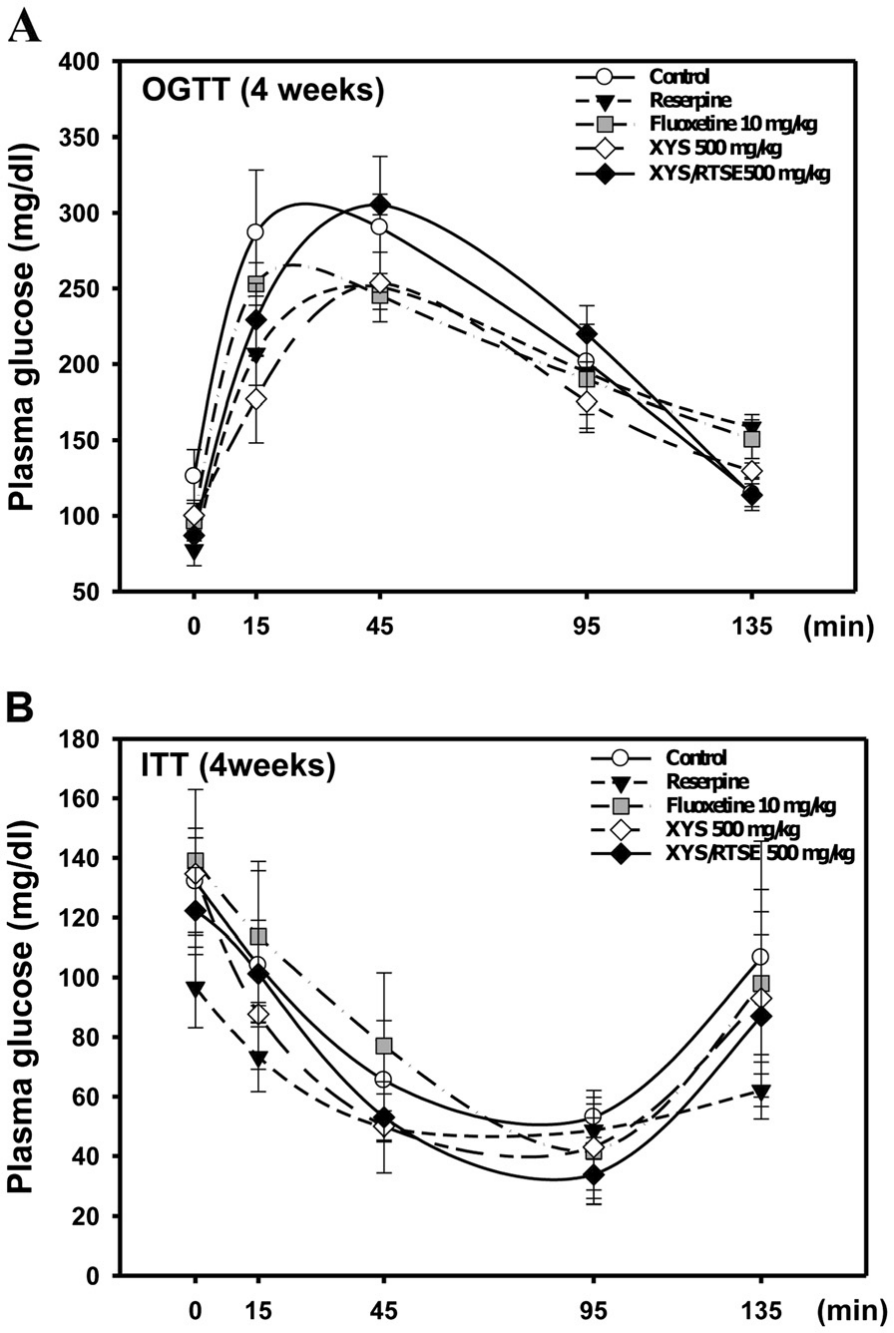
**Insulin and glucose dynamics.** C57BL/6 J mice (N = 8) were evaluated and compared for insulin secretion. After 4 weeks of treatment with fluoxetine, XYS, and XYS/RTSES, the OGTT was performed for the regulation of plasma glucose (**A**) and the ITT was performed for the plasma glucose response (**B**) after fasting for 16 h. Each value is expressed as the mean ± SD of 8 mice. *P < 0.05, **P < 0.01, ***P < 0.001, significant difference compared with control C57BL/6 J mice. ^#^*P* < 0.05, ^##^*P* < 0.01, ^###^*P* < 0.001, significant difference compared with XYS alone.

After pretreatment with reserpine (2 mg/kg/day) for 4 consecutive days, the treatments with XYS/RTSES, XYS, and fluoxetine were administered. The OGTT was monitored for 4 weeks. The fasting blood glucose levels were reduced in the 4 weeks reserpine-treated group in time-intervals (0 min: *P* = 0.02; 15 min: *P* = 0.06; 48 min: *P* = 0.341; 95 min: *P* = 0.78; 135 min: *P* = 0.0105) compared with the control group. The normal glucose tolerance curve did not show any significant differences compared with the control group throughout the study period after the administration of XYS/RTSES for 4 consecutive weeks. Neither XYS nor fluoxetine could reverse the reserpine-induced hypoglycaemia (Figure 4A).

The ITT was performed to evaluate glucose tolerance in depression induced by reserpine. After 4 weeks of treatment, the blood glucose levels were decreased in the XYS-treated (*P* = 0.22), XYS/RTSES-treated (*P* = 0.0066), and fluoxetine-treated (*P* = 0.013) groups compared with the reserpine-treated group (Figure 4B). The combined results from the OGTT and ITT suggest that XYS/RTSES increases the insulin sensitivity in reserpine-induced depression.

### Expression of 5-HT_1A_ receptors

**Figure 5:**
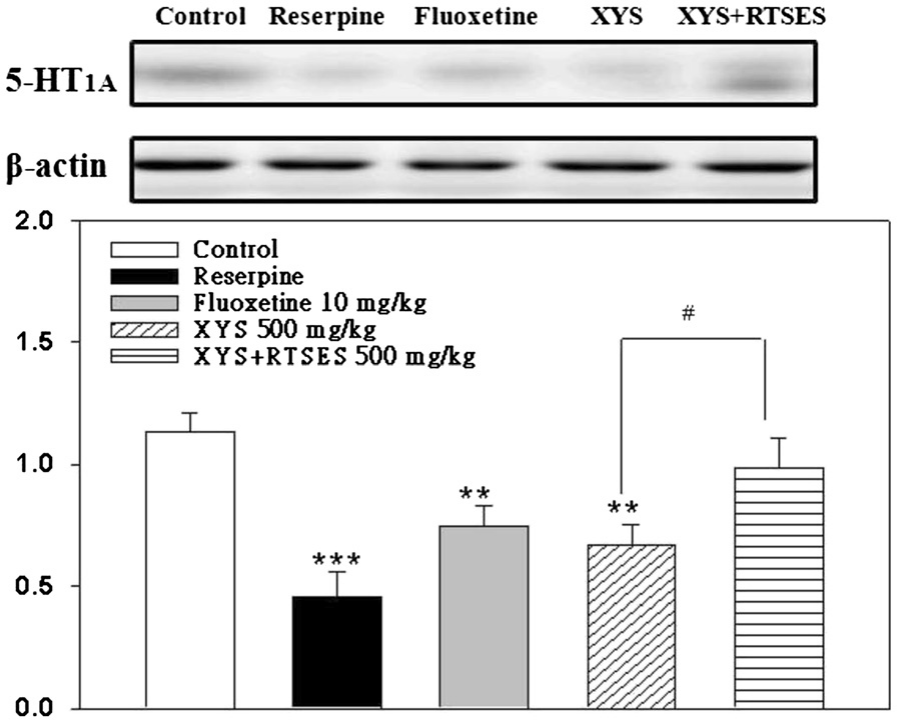
**Improvement of 5-HT**_**1A**_**receptor expression in the cerebrum.** (**A**) After 4 weeks of treatment with fluoxetine, XYS and XYS/RTSES, the 5-HT_1A_ receptor expression levels in C57BL/6 mice (N = 8) were detected by western blot analysis. (**B**) The β-actin levels were evaluated as a loading control, and the data are expressed as the 5-HT_1A_ protein/β-actin ratios (**B**). Each value is expressed as the mean ± SD of 8 mice. *P < 0.05, **P < 0.01, ***P < 0.001, significant difference compared with control C57BL/6 J mice. ^#^*P* < 0.05, ^##^*P* < 0.01, ^###^*P* < 0.001, significant difference compared with XYS alone.

A marked decrease in cerebral 5-HT_1A_ receptor expression was observed in the reserpine-treated group compared with the control group (*P* = 0.00084). The administration of XYS/RTSES for 4 consecutive weeks elevated the 5-HT_1A_ receptor expression (*P* = 0.0047) compared with administration of XYS or fluoxetine (Figure 5A), suggesting that the decreased 5-HT_1A_ receptor activity returned toward the basal level after XYS/RTSES treatment.

## Discussion

In this study, we established a reserpine-induced injury glial cell system and a depression-like mouse model to investigate the neuroprotective roles of XYS and XYS/RTSES treatments *in vitro* and *in vivo*. We strengthened the research of XYS for preclinical studies by reinforcing the extraction of the major active components and evaluating the potential pharmacological effects in an animal behavioural model through the RTSES.

In the glial cells, XYS/RTSES had a dose-dependent enhancing effect on cell proliferation. XYS/RTSES revealed partial neuroprotection compared with XYS. These data suggested that XYS/RTSES plays a pivotal role in controlling C6 glial cells and protects neurons against reserpine-induced oxidative stress, which is consistent with previous studies [38,39] that the release of glial cell line-derived neurotrophic factor from glial cells is a consequence of extracellular signal-regulated kinase/mitogen-activated protein kinase signalling activation by norquetiapine, which may contribute to the putative antidepressant properties of quetiapine.

In the EPM test, we found that oral administration of XYS/RTSES (500 mg/kg/day) for 2 consecutive weeks elevated the ratios of entries onto open arms to closed arms, compared with the same treatment with XYS (500 mg/kg/day) or fluoxetine (10 mg/kg/day). Meanwhile, XYS/RTSES also reversed the reduction in total entries in the reserpine-induced hypokinesia. However, the anxiolytic effects of XYS and fluoxetine were mostly blocked by reserpine pretreatment.

Some studies have identified that reserpine-induced hypokinesia was not reversed by either caffeine or trihexyphenidyl, which is a drug commonly used to reduce Parkinsonian symptoms [40,41]. Interestingly, fluoxetine has been demonstrated to increase activity levels and reduce immobility time in the FST, but decrease open-arm exploration in the EPM [38]. A similar study showed that treatment with fluoxetine (5-HT transporter) throughout mouse adolescence (3–7 weeks of age) did not produce detectable lasting abnormalities in either high-anxiety or low-anxiety inbred C57BL/6 J mouse strains [42]. These results suggest that XYS/RTSES could be a potential and prominent anxiolytic-like agent. Our FST and TST data demonstrated that XYS/RTSES administration significantly reduced the immobility time by 90%, while fluoxetine and XYS reduced the immobility time by 75% and 50%, respectively. The combined results suggest that XYS/RTSES may have anxiolytic- and antidepressant-like properties.

Consistently, rosiglitazone normalized hyperglycaemia, improved glucose tolerance, and significantly reduced the immobility time in the FST in db/db mice, suggesting an antidepressant-like effect [38,43]. Related studies demonstrated that 1 week of insulin treatment at 0.1 IU/g/day partially antagonized these depressive-like behaviours of streptozotocin-diabetic mice [44], while treatment with insulin at 1.0 IU/kg had a significant enhancing effect on the percentage of open-arm duration in anxious mice [45]. In accordance with these findings, XYS/RTSES improved the regulation of blood glucose and increased the insulin sensitivity in reserpine-induced glucose intolerance in mice, indicating that the antidepressant effect of XYS/RTSES may be partially caused by improvement of the reserpine-induced glucose intolerance.

Our OGTT data revealed that reserpine reduced the fasting blood glucose level at all time points (0–135 min). However, administration of XYS/RTSES for 4 consecutive weeks retained the glucose tolerance, while neither XYS nor fluoxetine reversed the reserpine-induced hypoglycaemia. 5-HT_1A_ receptor activation has been shown to increase dopamine release. As a potential mechanism, XYS/RTSES might increase the expression of 5-HT_1A_ receptor, and then the activation of D2 dopamine receptor induced a dose-related increase of plasma glucagons [46]. The ITT indicated that XYS/RTSES had an obvious effect on the variation in insulin sensitivity. Our results are consistent with previous studies demonstrating that 1 week of treatment with insulin at 0.1 IU/g/day partially antagonized these depressive-like behaviours in streptozotocin-diabetic mice on the Porsolt swim test [47], while treatment with insulin at 1.0 IU/kg significantly enhanced the effect on the percentage of open-arm duration in anxious mice on the EPM [48]. In accordance with these findings, XYS/RTSES improved the regulation of blood glucose and increased the insulin sensitivity in the reserpine-induced glucose intolerance in mice, indicating that the antidepressant effect of XYS/RTSES may be partially caused by the improvement in glucose intolerance.

Our results showed that reserpine decreased the 5-HT_1A_ receptor activity in the cerebrum, and confirmed that XYS/RTSES had antidepressant-like effects similar to typical antidepressants that were mediated by 5-HT_1A_ receptors. Our Western blotting data suggested the activation of cerebral 5-HT_1A_ receptors was responsible for the anxiolytic and antidepressant actions after XYS/RTSES treatment.

The action mechanisms of the anxiolytic- and antidepressant-like behavioural effects could be at least partly caused by the protection against reserpine-induced C6 glial cell damage and augmentation of the activation of cerebral 5-HT_1A_ receptors in reserpine-induced depressant-like mice.

The RTSES is simple, feasible, efficient, and suitable for highly valuable Chinese herbal medicines. Recent research has also shown that ultrasound-assisted extraction of the common Chinese herbal prescription *Xiao-chai-hu-tang* is feasible for replacing the traditional, time-consuming, and low-efficiency preparation procedures, and produces the highest bioactive constituent concentrations and best antioxidant functionalities [24].

## Conclusion

The XYS/RTSES significantly elevated the shuttle activity, reduced the immobility time on TST and FST, also improved the regulation of blood glucose and increased the insulin sensitivity in reserpine-induced anxiety and depression in mice. The activation of cerebral 5-HT_1A_ receptors and neuroprotection of glial cells may be involved in the mechanisms of XYS/RTSES actions. RTSES could provide a novel method for extracting the anxiolytic- and antidepressant-like substances from XYS.

## Abbreviations

XYS: *Xiao-Yao-San*; KSS: *Kami-Shoyo-San*; RTSES: Room-temperature super-extraction system; FST: Forced swimming test; TST: Tail suspension test; EPM: Elevated plus-maze; 5-HT_1A_: 5-hydroxytryptamine 1A; OGTT: Oral glucose tolerance test; ITT: Insulin tolerance test.

## Competing interests

The authors declare that they have no competing interests.

## Authors’ contributions

SHY conceived the study design and coordinated the study. JJC wrote the manuscript. WMWK, YLK, JCC, SLL, and CHC performed the animal experiments and biotechnological assays. WCK and HYW studied the animal behaviour. CCW, TJC, CYC, and KCL performed the statistical analyses. All authors read and approved the final manuscript.
